# Conflicting Views in Narratives on HIV Transmission via Medical Care

**DOI:** 10.1177/2325958218821961

**Published:** 2019-01-15

**Authors:** Michael A. Vance

**Affiliations:** 1 College of Pharmacy and Health Sciences, Butler University, Indianapolis, IN, USA

**Keywords:** Africa, AIDS, colonialism, Cuba, Haiti, HIV, HIV subtype B, iatrogenic

## Abstract

Molecular studies suggest that HIV arose in Africa between 1880 and 1940. During this period, there were campaigns by European colonial governments that involved unsterile injections of large numbers of Africans. That, along with other unsafe therapeutic interventions, may have propelled the evolution of HIV from SIV. Since subtype B in Africa may have been concentrated in white African homosexuals, it is possible that Westerners rather than Haitians introduced the virus to the New World. Amplification of HIV subtype B took place in Haiti, where transmission was facilitated by hazardous medical procedures including plasmapheresis. Representations in the media, however, largely ignore Western contributions to the spread of AIDS. This article focuses on the value of alternative narratives in fostering a balanced view that is less stigmatizing on developing nations.

**What Do We Already Know about This Topic?**

We know that HIV arose in Africa and that in the early phases of the pandemic medical interventions contributed to its spread.

**How Does Your Research Contribute to the Field?**

This article highlights evidence that the spread of HIV may differ from that which is commonly accepted.

**What Are Your Research’s Implications toward Theory, Practice, or Policy?**

The alternatives presented here will help diminish stigma associated with AIDS in some populations as well as encourage improved infection control measures.

## Introduction

According to the World Health Organization, around 35 million people have died of AIDS and nearly 1 in 25 adults in sub-Saharan Africa are currently infected with HIV, the virus that causes AIDS.^1^ Phylogenetic studies and other research on HIV/AIDS have generated histories on the origins of the pandemic. Such narratives, formulated by experts, inform policy and impact the lives of people throughout the world.

Genetic polymorphisms in infectious agents between populations demonstrates relatedness but determining the exact routes of evolution in time and space entails speculation. For a given set of circumstances (outcome), there may exist more than one potential pathway leading to that outcome. Acknowledging this uncertainty is especially important in a delicate matter such as a disease associated with sexuality, drug use, and poverty. HIV-positive victims among the economically disadvantaged have few advocates in the socially rarified world of biomedical science. It is critical to appreciate the potential negative impact on HIV-infected peoples of some of these narratives, such as perpetuating stereotypes of sexual promiscuity.^2^

The focus of this essay is the value of alternatives to 2 dominant narratives on HIV and AIDS: That Haiti was responsible for bringing HIV from Africa to the West and that injections and other invasive medical procedures have a negligible contribution to the spread of AIDS.

## Transmission of HIV

HIV passes from person to person by exchange of body fluids, which can occur iatrogenically and through nonmedical activities (Table 1). Because of differing environments and customs, including sexual practices, and dissimilarities in biology of HIV strains and human populations, HIV transmission varies geographically. Pattern 1 designates a “concentrated epidemic” in which HIV circulates in a limited subpopulation engaged in risky behavior, while pattern 2 is “generalized,” in which most victims are not identified with a high-risk subpopulaton.^3^ Pattern 1, which predominates in North America, Australia, and Western Europe, is spread by intravenous drug abuse and needle sharing and men having sex with men. With pattern 2, in sub-Saharan Africa and much of the Caribbean, heterosexual transmission is a major mode of infection.

**Table 1.** Medical and Nonmedical Transmission of HIV.

I. Iatrogenic transmission Blood and blood product transfusions and related activities Injections Other invasive procedures: surgery, dialysis, dental work, colonoscopy, and so on
II. Nonmedical transmission Intravenous drug abuse Homosexual relations Heterosexual relations Vertical transmission (mother to child in utero or breastfeeding) Tattoos, body piercing, scarifications, genital mutilation, and so on

Clarifying the underlying mechanisms for these patterns is a bit awkward. In the United States, for instance, the sexual revolution of the 1960s included heterosexuals, but heterosexual transmission there was low.

**Figure 1.**
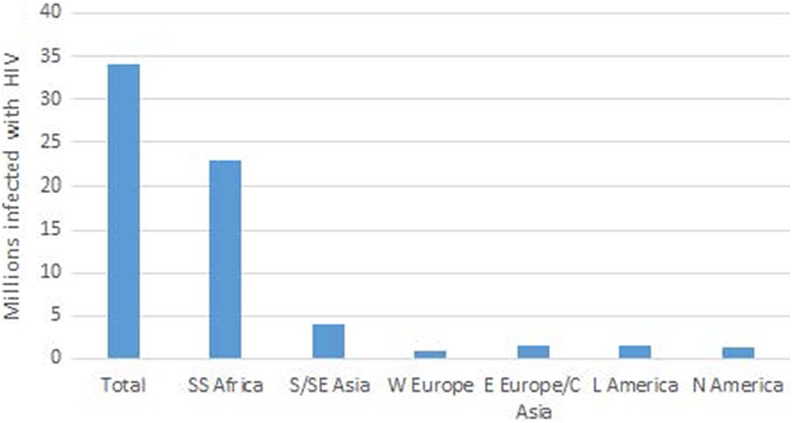
Millions of persons infected in different geographic regions (USAIDS World AIDS Day Report, 2010): Sub-Saharan Africa, South and Southeastern Asia, Western Europe, Eastern Europe and Central Asia, Latin America, and North America.

The prevalence of HIV infection in black Africa is staggeringly high (Figure 1), too high, it would seem, to be explained simply by transmission through heterosexual relations. Since it is widely persecuted and often illegal in sub-Saharan Africa, homosexual relations are probably more widespread than reported and hidden homosexual transmission might be misclassified as heterosexual. As an explanation for high prevalence of AIDS in Africa that doesn’t hold because in pattern 1, there is a preponderance of men with HIV while in African’s pattern 2 more females are infected than males. HIV passage between gay men involves anal intercourse and the prevalence of this practice among heterosexual Africans, because it is stigmatized, may also be underappreciated.^4,5^ If unprotected male–female anal intercourse is common, that would be an alternative explanation more consistent with observed sex ratios—especially considering that men who have sex with men are often married to women.^5^ It is also possible, but far from certain, that dry sex (drying and tightening of the vagina for sex, a common practice in some communities) enhances HIV transmission.^6^ Additionally, iatrogenic transmission, which would affect women more than men because they undergo more invasive procedures, may contribute to HIV burden in Africa.^7^

## Origin of AIDS

Although some have suggested a non-African origin,^8^ a persuasive argument has been made that the M group of HIV-1, the only HIV that has spread around the world, originated from SIVcpz, the SIV from chimpanzees in Central Africa.^9^ HIV-2, an immunosuppressive retrovirus endemic in Western Africa, is derived from SIVsmm that infects another primate, the sooty mangabey, in that region.^9^ Genetic analysis not only localizes the origin HIV-1M to Central Africa but also suggests that all HIV-1M traces back to a single SIVcpz and molecular clock studies, based upon the divergence of HIV-1 and SIV nucleic acid sequences, indicate that the jump to humans was recent with estimates ranging from 1880 to 1940.^10-12^

**Figure 2.**
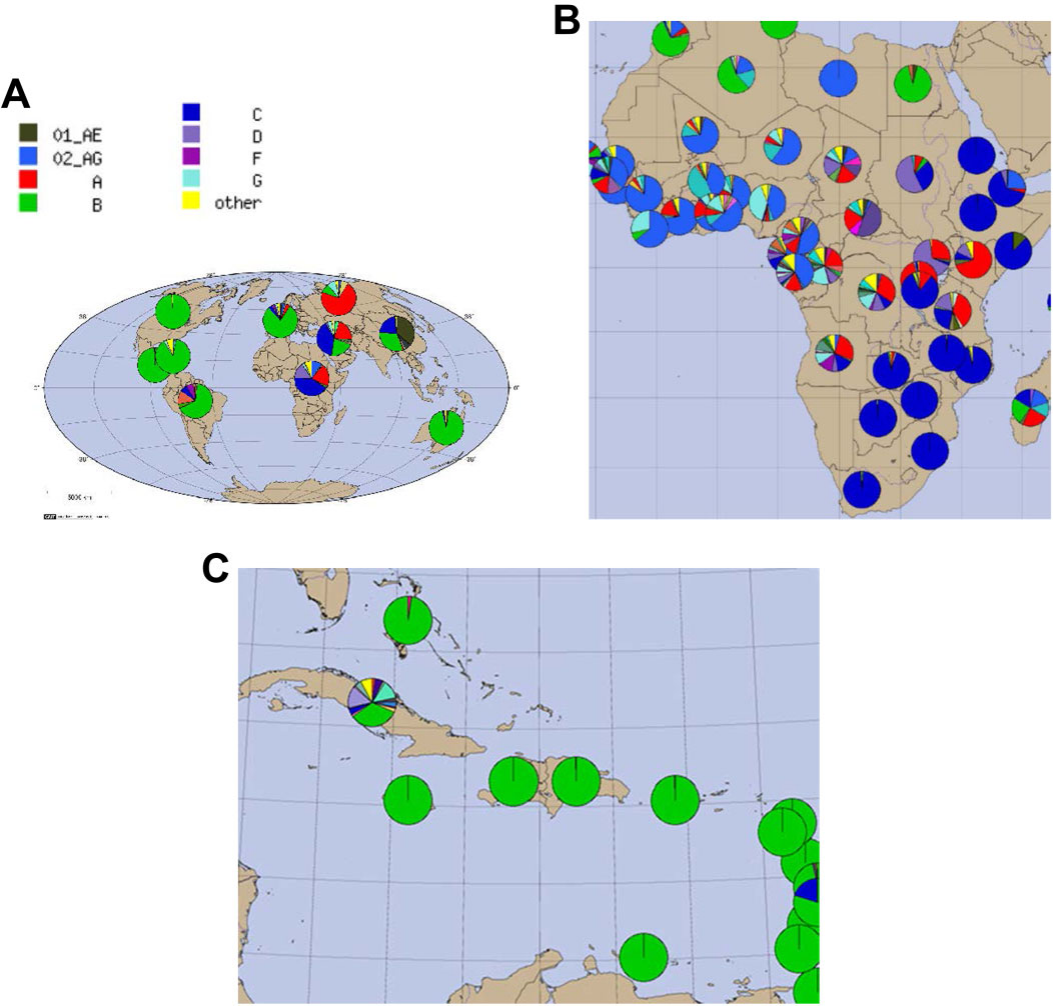
From Los Alamos National Laboratory, HIV sequence database (http://www.hiv.lanl.gov/components/sequence/HIV/geo/geo.comp). The frequency of HIV-1M subtypes by geographic areas according to the HIV sequence database. Subtypes with 2 letters refer to those derived from recombination of subtypes. A, Distribution of HIV-1M subtypes: World. B, Distribution of HIV-1M subtypes: Africa. C, Distribution of HIV-1M subtypes: Caribbean.

Over time, HIV-1M traveled to different locations where it evolved separately to form different branches of the phylogenetic tree—the different subtypes, or clades, of HIV-1M. In North America, South America, the Caribbean, Western Europe, and Australia, subtype B dominates over all others (Figure 2A-C). In sub-Saharan Africa, the most prevalent subtype of HIV-1M is C. Each of the major subtypes are present in Africa and Kinshasa, a Congo city considered an early focal point for the spread of HIV, not surprisingly, has all major subtypes circulating.^11^ It is curious, however, that subtype B, though relatively uncommon in Africa, came to dominate in so many regions outside that continent (Figure 2A).

Hunters who kill and butcher monkeys for bush meat mix simian blood (and other fluids) with their own and passage of SIV to humans in these circumstances is probable.^13^ SIVcpz infection of a hunter in the Central African range of infected chimpanzees is suspected to be the source of what was to become HIV-1M. But people have been hunting, butchering, and otherwise interacting with SIV-infected primates for millennia. What was different in the 20th century that allowed SIV to evolve in Africa and HIV to arise and create the AIDS pandemic?

One theory is that oral polio vaccine used in Central Africa in the 1950s and 1960s was contaminated with SIV due to alleged use of chimpanzee kidney cells in vaccine preparation.^14^ Millions of persons were injected, after all, with polio vaccine that had been tainted with a different virus, simian virus 40. Aside from the fact that the oral polio vaccination occurred too late—by the 1950s, AIDS was in Central Africa^11,15,16^—the supporting evidence is not strong.^16^ One reviewer of the polio theory stated,

I asked several AIDS researchers where they thought HIV came from. Just about all of them said they thought it was an old African disease that emerged from the bush when the winds of change blew through the continent—that HIV was driven by urbanization, war, trucking, mining and prostitution.^17(p17)^

A massive increase in population was part of an explosion of social changes that transformed Africa with the arrival of the European colonial period at the end of the 1800s.^18,19^ It is possible that population growth alone was sufficient for HIV to emerge in Africa, but other factors have worked synergistically to unleash AIDS: urbanization with infrastructure improvements paradoxically facilitating greater mobility and dissemination of disease,^19^ breakdown of traditional society, transmission by sex workers,^20^ untreated genital ulcers,^21^ lack of circumcision in men,^22^ other sexually transmitted diseases,^23^ and so on.

## Iatrogenesis and AIDS

In 18 months starting in 1917, French physician Eugene Jamot treated 5347 cases of African trypanosomiasis (sleeping sickness) with injections of atoxyl, a toxic arsenical, using 6 syringes.^7,24,25^ The subcutaneous atoxyl was often combined with intravenous therapy with either tartar emetic or tryparsamide.^24^ At the time, it was appreciated that patients occasionally contracted hepatitis after injections, but given the obliviousness to other dangers, it is near certain that adequate sterilization of needles and syringes did not occur.^25^

The first 1 to 2 months of an HIV infection is characterized by a high level of viremia and if SIV partially adopted to humans behaves similarly, that would create a “perfect storm” for the serial passage of SIV to ultimately produce a virus adapted to humans. The injections were given weekly for 12 weeks, so that a patient infected early could contaminate the syringes used for several weeks thereafter, when virus levels in the blood are high.

Animal infection with simian–human immunodeficiency virus (SHIV) supports the injection hypothesis. The SHIV, an engineered virus, did not cause illness in the monkeys infected nor was it transmissible to other monkeys in the colony. But serial passage in macaques, through intravenous injection and bone marrow transplant, generated a virulent virus that caused an immunodeficiency disease that passed from monkey to monkey.^26,27^

A single campaign using inadequately sterilized injection equipment in Central Africa seems insufficient to account for the emergence of HIV/AIDS. The question is, therefore, whether unsafe injections, transfusions, and medical procedures were commonplace during the critical period of the late 1800s and first half of the 1900s. The answer is a resounding “yes,” to the extent that Drucker et al^25^ labeled the era the “century of the injection” (Table 2). The smallpox campaign involved intradermal injections but that does not necessarily imply low risk. Due to lack of refrigeration in hot climate, the vaccinia virus was maintained in chains of humans—pus from one vaccinated person to vaccinated others who, in turn, donated their pus for more vaccines.^28^

**Table 2.** Major Colonial Era Undertakings with Potential for Iatrogenic Transmission of Blood-Borne Viruses in Central Africa. Disease Treatments All Involved Injections.

Dates	Project	References
1893-1910	Up to 35 000 smallpox arm-to-arm vaccinations	^25^
1920-1935	Massive French anti-sleeping sickness campaign with from 60 000 to 600 000 injections per year	^24,28^
1930s-1950s	Campaigns undertaken against yaws, syphilis, kala-azar, and leprosy	^24,30^
Late 1940s-1954	Tens or hundreds of thousands of injections yearly in Leopoldville STD clinics. Many received injections prophylactically or with misdiagnosed as having STDs	^24,33^
1950s-1970s	Large-scale polio, yellow fever, and smallpox vaccinations. After 1960, oral polio vaccine took the place of injections	^30^
1940s forward	Widespread use of transfusions to treat anemia of malaria, pregnancy, sickle cell, and other diseases	^29^

There have been at least 2 books^7,24^ and numerous articles^25,29-32^ assembling a reasoned argument that injections, transfusions, and other medical procedures introduced by colonial powers facilitated the emergence of AIDS. Once established, unsafe medical procedures may have accelerated virus expansion.^32,33^

The injection theory of the genesis of HIV is impossible to discard. Yet it has been easy to overlook, sometimes escaping significant mention even in works that fault colonialism for establishing conditions favoring the diffusion of AIDS.^8,34,35^ Schneider and Drucker said,

It is remarkable that despite so much evidence of the wide use of blood transfusions during the decades before the emergence of HIV in sub-Saharan Africa, there is almost a total absence of discussion of these millions of transfusions for the beginning of the AIDS epidemic in Africa…European countries were eager to introduce the new techniques developed during World War II to demonstrate the value of Western technology that accompanied colonial rule.^29(p992-993)^

The same eagerness holds for the modern hypodermic syringe itself, though it was perfected independently in 1853 by Alexander Wood and Gabriel Pravaz. By the later 1800s, the syringe had become wildly popular in the West. Its use for intravenous injection of narcotic analgesics created legions of addicts but that apparently didn’t dampen the enthusiasm of colonists to use this new technology (Table 2).

As to why AIDS is so astonishingly common in sub-Saharan Africa (Figure 1), many explanations have been proffered. Several factors likely operated simultaneously to facilitate the transmission of HIV in sub-Saharan Africa and their interactions may not be straightforward. Controlled trials demonstrate that male circumcision, for example, has a protective effect against sexual transmission of HIV,^36^ but encouraging it could be counterproductive if operations are performed with unsterilized instruments.

Prominent is the “concurrency hypothesis” that AIDS is prevalent in Africa because Africans, allegedly more so than other peoples, carry on simultaneous relationships with more than one sexual partner.^34,37,38^ That this type of heterosexual networking is driving the AIDS epidemic is now sometimes stated as fact even though there are observations that cast doubt on it.^39,40^

Early in the AIDS pandemic official sources stated that iatrogenesis was an important driver of HIV infection in sub-Saharan Africa. The later turnaround, discounting medical spread and blaming sexual transmission nearly exclusively, seems grounded in proof by forceful repetition. Duh’s description of health care in sub-Saharan Africa suggests a potential for underestimating iatrogenic transmission.

There is a widespread belief in many African countries that injected medication is more effective than oral medication. Patients (or parents of pediatric patients) tend to be disappointed when a clinic issues oral medication instead of an injection. Some people even request an injection if it is not offered…. The use of injections in many African countries is not limited to clinics and hospitals. Private individuals, particularly in rural areas, may keep some medication and injection equipment in their homes to give injections to town folks. In some cases, people giving injections are inadequately trained in aseptic techniques.

I visited a rather large clinic in a big city in Africa in December 1987. In a conversation with one of the clinic doctors, she remarked that the doctors in the country were fully aware of the potential danger of reusing disposable needles, ‘but we cannot, we simply cannot afford to throw needles and syringes away’.^41(p1856)^

Assertions that iatrogenic AIDS is rare in sub-Saharan Africa based upon computer modeling are disconcerting for several reasons. Estimates of the probability of infection from injection with contaminated equipment have been based, in part, on data from accidental needle sticks which may underestimate risk, since in such cases the plunger is not depressed.^42^ Reuse of needles and syringes may be concealed and therefore underrepresented in the models,^43,44^ the prevalence of injections by untrained providers is likewise underappreciated along with the demand for injections^41,43^ and iatrogenic spread through transfusions and invasive procedures.^45,46^ Medical transmission of HIV is misclassified as heterosexual spread when the risk of the latter is exaggerated due to a variety of reasons, including not taking into consideration the decrease in frequency of coitus with at-risk partners that accompanies concurrency,^47^ and an assumption that seroconversion is always due to sexual transmission.

Contracting HIV is more likely in Africa simply because a greater percentage of the population is HIV positive, increasing the likelihood that a sexual encounter or dirty syringe includes the virus. Beyond that, access to antiretroviral drugs is less, which means that blood and secretions are more infective and prophylactic use in at-risk persons is limited. Infectivity of HIV in the West is not necessarily the same as African strains of HIV are different and Africans, with poorer nutrition and chronic diseases like schistosomiasis and malaria, may be more vulnerable to infection. Estimates of the efficacy of HIV transmission through injections from experience with Western populations do not reliably reflect the situation in other cultures. It is not improbable, based on the reality of health care in resource poor countries, that medical transmission of HIV is substantial, at least in some localities.^48^

## Haiti

In 2007, the popular press announced that scientists had demonstrated that HIV-1 subtype B was first carried by Haitians from Africa to Haiti, from where it spread to the rest of the world.^49^ The reports were based on a study of HIV genomic sequences from Haitians and patients from other countries to find that the oldest, non-African HIV subtype B was in Haiti, “the most parsimonious explanation for this pattern is that all those subtype B infections across the world emanated from a single founder event linked to Haiti.”^50(p1856)^ The article generated objections from Haitians, who felt unfairly blamed, and rebuttals from scientists in agreement.^51^ While the Haiti to US spread seems probable,^52^ to assert it with certainty dismisses viable alternatives.

It is not impossible that North Americans or Western Europeans carried subtype B to their home country where conditions for its propagation were lacking. Westerners could then have brought the disease to Haiti, through sexual tourism or other route, and if circumstances were favorable, amplification could have occurred there while the original HIV subtype B met a dead end in the Western country.

Conditions in Haiti were indeed favorable for amplification of HIV infection whether introduced from Africa or the West. Hemo-Caribbean was open for 20 months in 1971 and 1972 in Port-au-Prince, the capital of Haiti. It was a center for plasmapheresis where desperately poor Haitians sold their plasma for a few dollars.^24^ Foreign businesses in turn purchased the plasma for production of antibodies, clotting factors, and other products; at its peak, some 6000 L/mo were shipped to American pharmaceutical companies.^53,54^ The formed elements were reinfused in the Haitian donor so that, unlike blood donations, a person could sell plasma several times a month. The operation paid inadequate attention to the safety of the donors and to screening of plasma for diseases^53^ and, combined with the deficiency in sterile supplies and techniques characteristic of health-care delivery in impoverished lands, that would create another “perfect storm” for HIV spread.

There are issues with that alternative scenario. Subsequent phylogenetic studies of HIV are consistent with an earlier Caribbean epidemic.^55^ Using viral RNA recovered from Haitian and US patients in the 1970s, Worobey et al^55^ estimated a growth rate of HIV as rapid or more rapid in the United States compared to Haiti. That suggests that conditions in the United States, at least in the gay community, were favorable to the propagation of HIV. Sex tourism flourished in Haiti in the late 1970s but was less in the 1960s, the decade when HIV seems to have been introduced into the New World. Thousands of Haitian workers were sent to Zaire, the newly liberated Belgian Congo, after its independence in 1960, so there was great opportunity for them to bring the virus back home.

The peculiar distribution of HIV-1 subtypes in South Africa, however, is consistent with primary spread to the West and secondary transport to Haiti.

## South Africa

As in other sub-Saharan countries, HIV-1 subtype B is uncommon in South Africa. In 1997, van Harmelen et al^56^ reported, however, that of 26 homosexuals with HIV infection, 25 had subtype B while only 4 of 32 heterosexuals did. More telling, the homosexual group was 62% white and had no blacks while heterosexuals were 66% black and 9% white. A larger South African study, limited to men who have sex with men, subsequently confirmed that white men were usually infected with subtype B and black men almost always with subtype C.^57^ Though AIDS has hit South Africa very hard, it was a latecomer, with the first 2 cases described in the literature in 1983.^58^ They were both homosexual flight attendants who presented with full-blown AIDS, including diarrhea and weight loss. Given the lag time to symptoms in HIV infection, they probably acquired HIV in the early 1970s. Both had traveled to the United States and Europe.

While it is sometimes assumed that gay white Africans contracted HIV from sexual contact with European or American men, an alternative infection source helps explain the unusual B subtype global distribution. Subtype B evolved separately in Africa among a privileged, jet-setting, homosexual, largely white population. Sexual encounters of this group with Europeans and North Americans were common, facilitating a spread to these continents prior to the Caribbean.^59^ Although infected Westerners brought HIV with them wherever they went, it was only when it was introduced to Haiti, primed by poverty for iatrogenic spread, that the disease took off.

## Cuba

The epidemiology of HIV in Cuba is strikingly different from Haiti and the rest of the Caribbean and the Americas. This Cuban anomaly bears on how HIV disseminated.

In most Caribbean countries, HIV-1 subtype B infections dwarfs the others but in Cuba subtype B represents a small fraction of HIV infections and, as in Central Africa, a wide variety of HIV-1 subtypes are represented (Figure 2C). This is predictable. From the revolution of 1959 until after the fall of the Soviet Union in 1991, Cuba military and civilian personnel were stationed in or near HIV-afflicted countries in Africa while persons from these countries spent long periods studying and training in Cuba. There was also considerable exchange between Cuba and Eastern Europe, but that area is believed to have been relatively free of AIDS until the 1990s.

**Figure 3.**
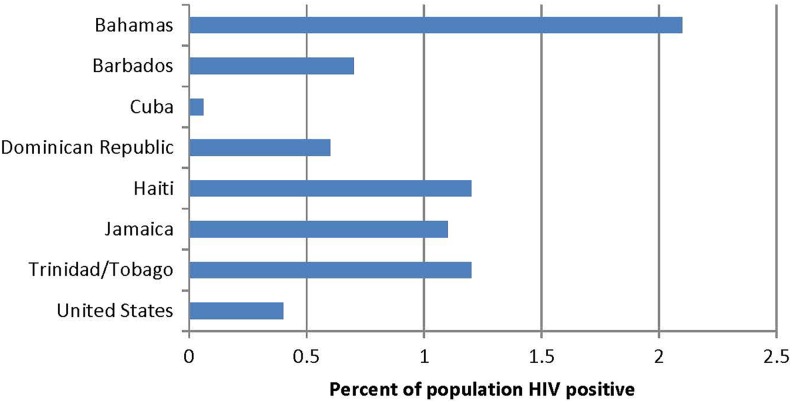
Estimated percentages of population with HIV in Caribbean countries and the United States. Percentages calculated from people living with AIDS in the UNAIDS 2010 Global Report (http://www.unaids.org/globalreport/Global_report.htm) divided estimated population in 2010.

Despite repeated introduction of the retrovirus into Cuba, as demonstrated by the diversity of HIV strains, the prevalence of infection there is radically lower than other countries in the region (Figure 3). The rarity of HIV/AIDS in Cuba has been credited to its policy of forced quarantining HIV-positive individuals and its aggressive screening, treatment, and follow-up of cases. These policies, however, could not be instituted until a blood test was available to Cuba in 1986. By rights, AIDS should have by then been a devastating plague in Cuba as it was in Haiti. There is something about Cuba that was operational before HIV testing that prevented AIDS from gaining a foothold (Table 3).

**Table 3.** Conditions in Cuba That Favor and Conditions That Deter HIV Transmission.

Conditions that Favor	Conditions that Deter
Concurrent multiple partners Commercial sex workers (but less common early in the revolution) Men not circumcised Much international travel and exchange with countries with high prevalence of HIV infection	Little intravenous drug abuse Condoms now widely accepted (but perhaps not before public health condom campaign in response to AIDS threat) Public well educated about AIDS Government quarantined the HIV positive (in past) Women perhaps more empowered in Cuba Health care in Cuba discourages iatrogenic transmission

If the medical system of Cuba were of the standard of other poor countries, it is unlikely that AIDS would be uncommon. Cuban medicine is delivered by highly trained professionals who are cognizant of the importance of aseptic technique. The low prevalence of HIV in Cuba reflects a powerful effect of controlling iatrogenic spread.

## Conclusion

Many times in Italy I laughed, hearing the Italians speak of the French disease, and the French call it the disease of Naples; and in truth both would have had the name better if they had called it the disease of the Indies.^60(p72)^

This famous quote concerning syphilis illustrates a universal human attribute of, blaming the other, especially in things sexual. Why AIDS suddenly surfaced in the 20th century is not, and likely will never be, known with certainty, but it is natural that the medical establishment would be reluctant to recognize medicine’s possible contribution. Hence, the ready acceptance of Africans’ sexuality as the driver of the AIDS epidemic.

Sexual transmission is important in spreading AIDS in Africa,^38,61,62^ but that does not mean it is the only driver and infection should not automatically be attributed to promiscuity or concurrent relations. There are factors other than promiscuity that facilitate conversion to HIV-positive status: untreated sexually transmitted infections and genital ulcers, lack of male circumcision, female genital mutilation, underuse of condoms, women not empowered to resist unprotected relations, men isolated from women by labor demands fomenting high-risk prostitution, and potential misrepresentation of iatrogenic HIV infection as sexual.^30,63-67^

The emergence of AIDS provides lessons that can be useful. One is that unthinking enthusiasm for new technology carries unanticipated risks,^30^ a lesson familiar to those versed in the history of pharmacotherapy. Another should be humility. That well-intentioned medical services rendered to African countries possibly created the AIDS pandemic should give us pause.

Reasoned arguments against a large role for medical transmission have been presented.^61,62,68,69^ Genotyping of HIV RNA indicates that, in one area of South Africa, sexual liaisons with older men explain the high rate of infections in young women.^70^ South Africa has the highest prevalence of HIV, yet its health care system is recognized as one of the continent’s best, suggesting that the contribution of iatrogenic transmission is modest. There are too many contradictory observations, however, to be disregarded: unexplained HIV-positive youngsters born of HIV-negative women, widespread use of unsterile needles, equipment, and transfusions; and the history of large outbreaks of nosocomial HIV infections under circumstances resembling those in Africa.^43,71-78^

War and political instability, characteristic of contemporary Africa, compromise controls over the quality of professional medical care and that provided by traditional practitioners. In resource-limited countries, this disruption can lead to outbreaks of iatrogenic infection. Such was the case in the 1980s in Romania, where many children in orphanages became HIV positive because of unsafe medical injections.^75^

A leading medical journal editorialized, “Although interest in how the outbreak originated may be a matter of scientific curiosity for the future, apportioning blame for the outbreak now is neither fair to people working to improve a dire situation, nor helpful in combating the disease.”^79(p813)^

That was not to defend Africans or Haitians being scapegoated for AIDS. It objected that the United Nations was held responsible—accurately, it turns out—for having introduced cholera to Haiti in the wake of the 2010 earthquake.

The possibility that modern medicine, a pride of industrialized societies, has been instrumental in spreading the AIDS pandemic is inherently onerous to decedents of colonialists. More than that, it conflicts with the preferred narrative: Colonialism was bad, but it did bring benefits like Western-style health care with hospitals and clinics that cure terrible diseases and eradicated or nearly eradicated smallpox, polio, and Guinea worm. It is more reassuring to implicate Africans and Haitians who do not have the sway of the United Nations.^80^

Ioannidis emphasized, in a highly cited article, that published research findings are frequently erroneous.^81^ Among his corollaries is that the hotter the field of research, the greater the likelihood that findings are false. Recognition of uncertainty is particularly important in the red-hot research area of AIDS. The real reason that AIDS is so prevalent in sub-Saharan Africa, and indeed some populations within the United States,^82^ is poverty and disenfranchisement.

In a communication that was forcefully disputed,^83^ Gisselquist asserted,

The hypothesis that HIV-positive wives are almost certainly infected via sex threatens millions of women with disgrace, divorce, loss of access to their children, and bodily harm. An alternative message—that HIV infections are not a reliable sign of sexual behavior—is not only mandated by the evidence, but also protects women and families.^84(p659)^

With regional differences in sexual and medical practices (not to mention biology), it is risky to definitively implicate one or the other as the driver of HIV infection for a single locale much less than for sub-Saharan Africa as a whole. The inability of mathematical models to account for dramatic changes of HIV infection rates in some African countries illustrates this.^85^ Recognizing uncertainty in our narratives promotes open-mindedness and minimizes stigmatization. In the present example, dismissing iatrogenic transmission of HIV not only harms women but also unnecessarily labels people as promiscuous.
